# Supplementary material for: Population homogeneity for the antibody response to COVID-19 BNT162b2/Comirnaty vaccine is only reached after the second dose across all adult age ranges
Source: Nat Commun. 2022 Jan 10;13:140. doi: 10.1038/s41467-021-27761-z (PMC8748650; doi:10.1038/s41467-021-27761-z)
Supplement: Supplementary file 1 — Supplementary Information [file 41467_2021_27761_MOESM1_ESM.pdf]

Population homogeneity for the antibody response to COVID-19 BNT162b2 / Comirnaty vaccine is only reached after the second dose across all adult age ranges.

Inventory of supporting information

Pdf file containing:

- **Supplemental References** – Reference list for R, R studio, and main package ggplot2 used for data management, graphical design, and statistical analysis (described in Methods section)
- **Supplemental Table 1**- Median and [IQR] for all semi-quantitative data on Ig levels presented in Figures 2-5
- **Supplemental Table 2**- Anti-Spike IgG Positivity in HCW and NHR as in **Figure 2** and stratified by age and sex as in Figure 4
- **Supplemental Table 3**- Median and [IQR] for ECLIA and ELISA data, and corresponding anti-spike IgG positivity at each time point, for HCW participants who contributed samples only at 1 or 2 of the 3 collection times (t0 only, t0 and t1, or t0 and t2)
- **Supplementary Figure 1**- Effect of delayed administration of 2nd vaccine dose in the NHR cohort
- **Supplementary Figure 2**- Infections after the 1st dose.

Supplementary References

Software for data management, graphical design, and statistical analysis

- R, version 4.0.4; GUI 1.74 *(R Core Team (2021). R: A language and environment for statisticalcomputing. R Foundation for Statistical Computing, Vienna, Austria. URL* https://www.R-project.org/*.)*
- RStudio, version 1.1.463; (*RStudio Team (2020). RStudio: Integrated Development for R. RStudio, PBC, Boston, MA URL* http://www.rstudio.com/*.)*
- R package ggplot2, (version 3.3.5); *H. Wickham. ggplot2: Elegant Graphics for Data Analysis. Springer-Verlag New York, 2016. https://ggplot2.tidyverse.org*

Supplemental Table 1. Median and [IQR] for all semi-quantitative data presented in **Figures 2-5**.

	Anti-N	Age	n	t0	t1	t2
HCW Anti-RBD Ig	neg	19-69	948	0.39 [0.39-0.39]	63 [23-138]	2207 [1238-2500]
		19-29	171	0.39 [0.39-0.39]	112 [47-208]	2500 [1887-2500]
		30-39	251	0.39 [0.39-0.39]	72 [40-157]	2393 [1525-2500]
		40-49	204	0.39 [0.39-0.39]	51 [17-127]	1971 [1171-2500]
		50-59	216	0.39 [0.39-0.39]	46 [14-102]	1921 [1075-2500]
		60-69	107	0.39 [0.39-0.39]	28 [12-85]	1599 [997-2500]
	pos	24-66	23	35.0 [5.05- 183.9]	2500 [2500-2500]*	2500 [2500-2500]*
HCW Anti-S IgG	neg	19-69	948	0.18 [0.15-0.23]	1.44 [1.26-1.56]	1.83 [1.72-1.92]
		M	197	0.18 [0.15-0.22]	1.42 [1.15-1.54]	1.82 [1.72-1.90]
		F	751	0.17 [0.15-0.23]	1.44 [1.27-1.57]	1.83 [1.72-1.92]
		19-29	171	0.19 [0.16-0.23]	1.54 [1.40-1.62]	1.87 [1.75-1.94]
		M	31	0.21 [0.17-0.30]	1.49 [1.35-1.58]	1.89 [1.73-1.94]
		F	140	0.18 [0.16-0.22]	1.55 [1.41-1.62]	1.86 [1.76-1.94]
		30-39	251	0.17 [0.15-0.22]	1.48 [1.34-1.58]	1.83 [1.72-1.92]
		M	51	0.18 [0.15-0.21]	1.49 [1.29-1.57]	1.81 [1.72-1.87]
		F	200	0.17 [0.15-0.22]	1.47 [1.36-1.58]	1.85 [1.72-1.92]
		40-49	204	0.17 [0.14-0.23]	1.40 [1.17-1.56]	1.83 [1.73-1.90]
		M	41	0.16 [0.14-0.21]	1.34 [1.13-1.49]	1.81 [1.69-1.87]
		F	163	0.17 [0.14-0.23]	1.41 [1.17-1.56]	1.83 [1.74-1.91]
		50-59	216	0.18 [0.15-0.23]	1.36 [1.16-1.49]	1.83 [1.70-1.90]
		M	45	0.18 [0.15-0.23]	1.35 [1.07-1.46]	1.85 [1.74-1.91]
		F	171	0.17 [0.15-0.23]	1.37 [1.17-1.49]	1.83 [1.70-1.89]
		60-69	107	0.17 [0.15-0.23]	1.30 [1.00-1.53]	1.81 [1.71-1.89]
		M	29	0.18 [0.15-0.21]	1.03 [0.63-1.49]	1.83 [1.74-1.89]
		F	77	0.17 [0.15-0.24]	1.36 [1.21-1.56]	1.79 [1.70-1.89]
	pos	24-66	23	0.94 [0.61-1.41]	1.85 [1.72-1.95]	1.91 [1.80-2.02]
NHR Anti-S IgG	n/a	70-99	118	0.20 [0.16-0.26]	0.50 [0.35-1.06]	1.83 [1.68-1.96]
		M	31	0.21 [0.16-0.26]	0.48 [0.36-1.16]	1.80 [1.62-1.84]
		F	87	0.20 [0.16-0.27]	0.52 [0.34-0.91]	1.85 [1.71-1.98]
		70-85	51	0.20 [0.16-0.27]	0.53 [0.42-1.21]	1.83 [1.69-1.91]
		M	18	0.23 [0.17-0.26]	0.51 [0.46-1.26]	1.81 [1.64-1.88]
		F	33	0.19 [0.16-0.28]	0.53 [0.41-1.18]	1.83 [1.69-1.94]
		86-99	66	0.20 [0.16-0.26]	0.46 [0.30-0.85]	1.84 [1.69, 1.97]
		M	13	0.20 [0.16-0.25]	0.36 [0.26-0.95]	1.78 [1.63-1.81]
		F	53	0.21 [0.16-0.26]	0.48 [0.31-0.79]	1.87 [1.72-1.98]
HCW Anti-S IgM	neg	19-69	949	0.62 [0.47-0.83]	0.92 [0.70-1.20]	0.95 [0.71-1.28]
		19-29	173	0.69 [0.51-0.89]	0.99 [0.83-1.35]	1.03 [0.78-1.32]
		30-39	251	0.68 [0.54-0.89]	0.99 [0.90-1.27]	0.98 [0.75-1.31]
		40-49	203	0.64 [0.46-0.86]	0.91 [0.68-1.17]	0.93 [0.70-1.31]
		50-59	217	0.56 [0.43-0.74]	0.86 [0.66-1.12]	0.90 [0.65-1.20]
		60-69	107	0.50 [0.39-0.66]	0.72 [0.54-0.98]	0.85 [0.58-1.24]
	pos	24-66	23	0.87 [0.63-1.07]	0.99 [0.75-1.41]	0.82 [0.73-1.18]
NHR Anti-S IgM	n/a	70-99	118	0.43 [0.30-0.53]	0.36 [0.24-0.48]	0.39 [0.26-0.58]
		70-85	51	0.42 [0.32-0.51]	0.36 [0.26-0.48]	0.39 [0.28-0.48]
		86-99	66	0.43 [0.28-0.56]	0.34 [0.22-0.48]	0.40 [0.24-0.65]
HCW Anti-S IgA	neg	19-69	949	0.45 [0.33-0.63]	1.11 [0.91-1.23]	1.08 [0.99-1.19]
		19-29	173	0.43 [0.30-0.59]	1.11 [0.93-1.24]	1.07 [0.94-1.17]
		30-39	251	0.45 [0.35-0.62]	1.12 [0.97-1.24]	1.07 [0.99-1.16]
		40-49	203	0.47 [0.34-0.61]	1.13 [0.90-1.24]	1.09 [0.98-1.22]
		50-59	217	0.45 [0.33-0.65]	1.08 [0.86-1.22]	1.10 [1.01-1.20]
		60-69	107	0.45 [0.32-0.65]	1.11 [0.88-1.20]	1.10 [1.04-1.27]
	pos	24-66	23	0.74 [0.60-0.95]	1.54 [1.30 1.95]	1.33 [1.10 1.61]
NHR Anti-S IgA	n/a	70-99	118	0.63 [0.44-0.86]	0.81 [0.53-1.09]	1.13 [0.96-1.39]
		70-85	51	0.64 [0.47-0.89]	0.82 [0.59-1.12]	1.15 [1.02-1.36]
		86-99	66	0.62 [0.43-0.80]	0.75 [0.50-1.07]	1.12 [0.94-1.37]

* When determined on 1/50 dilution of samples, t1 10743 [7560-15838], t2 15341 [8378-23593]*

Supplemental Table 2. Anti-Spike IgG Positivity in HCW and NHR as in **Figure 2** and stratified by age and sex as in **Figure 4**

	Anti-N	Age	n	Positivity; n (%; 95% CI) t1	t2
HCW	neg	19-69	948	845 (89.1;86.9-91.0)	947 (99.9; 99.3-100.0)
		M	197	166 (84.3; 78.4-88.7)	196 (99.5; 96.4-99.9)
		F	751	679 (90.4; 88.1-92.3)	751 (100; 99.5-100)
		19-29	171	168 (98.2; 94.6-99.4)	171 (100; 97.8-100)
		M	31	30 (96.8; 78.4-99.6)	31 (100; 89.0-100)
		F	140	138 (98.6; 94,4-99.6)	140 (100; 97.3-100)
		30-39	251	241 (96; 92.7-97.9)	251 (100; 98.5-100)
		M	51	50 (98; 86.6-99.7)	51 (100; 93.0-100)
		F	200	191 (95.5; 91.5-97.7)	200 (100; 98.1-100)
		40-49	204	175 (85.8; 80.2-90.0)	204 (100; 98.1-100)
		M	41	33 (80.5; 64.8-90.2)	41 (100; 91.4.100)
		F	163	142 (87.1; 81.0-91.5)	163 (100; 97.7-100)
		50-59	216	182 (84.3; 78.7-88.6)	215 (99.5; 96.7-99.9)
		M	45	37 (82.2; 67.6-91.1)	44 (97.8; 84.9-99.7)
		F	171	145 (84.8; 78.5-89.5)	171 (100; 97.8-100)
		60-69	106	79 (74.5; 65.2-82.0)	106 (100; 96.5-100)
		M	29	16 (55.2; 36.1-72.86)	29 (100; 88.3-100)
		F	77	63 (81.8; 71.3-89.0)	77 (100; 95.3.100)
	pos	24-66	23	22 (95.7; 79.0-99.2)	23 (100; 85.7-100)
NHR	n/a	70-99	118	30 (25.4; 18.4-34.0)	112 (94.9; 89.4-98.0)
		M	31	9 (29; 16.1-46.6)	28 (90.3; 75.1-96.7)
		F	87	21 (24.1; 16.4-34.1)	84 (96.6; 90.3-98.8)
		70-85	51	15 (29.4; 18.3-43.7)	49 (96.1; 85.0-99.1)
		M	18	6 (33.3; 14.4-59.7)	17 (94.4; 64.5-99.4)
		F	33	9 (27.3; 14.3-20.3)	32 (97.0; 80.0-99.6)
		86-99	66	14 (25.4; 18.4-34.0)	62 (93.4; 84.6-97.8)
		M	13	3 (23.1; 6.3-57.2)	11 (84.6; 49.0-96.9)
		F	53	11 (20.8; 11.7-34.2)	51 (96.2; 85.6-99.1)

Supplemental Table 3. Median and [IQR] for ECLIA and ELISA data, and corresponding anti-spike IgG positivity at each time point, for HCW participants who contributed samples only at 1 or 2 of the 3 collection times (t0 only, t0 and t1, or t0 and t2).

	t0 (n=220)	t1 (n=79)	t2 (n=61)
**median [IQR]**			
Anti-RBD Ig	0.39 [0.39-0.39]	75.72 [34.66-152.55]	2500 [1355-2500]
Anti-S IgG	0.18 [0.15-0.23]	1.47 [1.35-1.56]	1.79 [1.72-1.93]
Anti-S IgM	0.67 [0.52-0.86]	0.96 [0.72-1.22]	0.88 [0.69-1.17]
Anti-S IgA	0.48 [0.35-0.69]	1.16 [1.01-1.31]	1.09 [0.93-1.15]
**Positivity; n (%; 95 CI)**			
Anti-S IgG	9 (4.1%; 2.2-7.6)	77 (97.5%; 91.2-99.3)	61 (98.4%; 91.3-99.7)

Supplementary Figure S1.

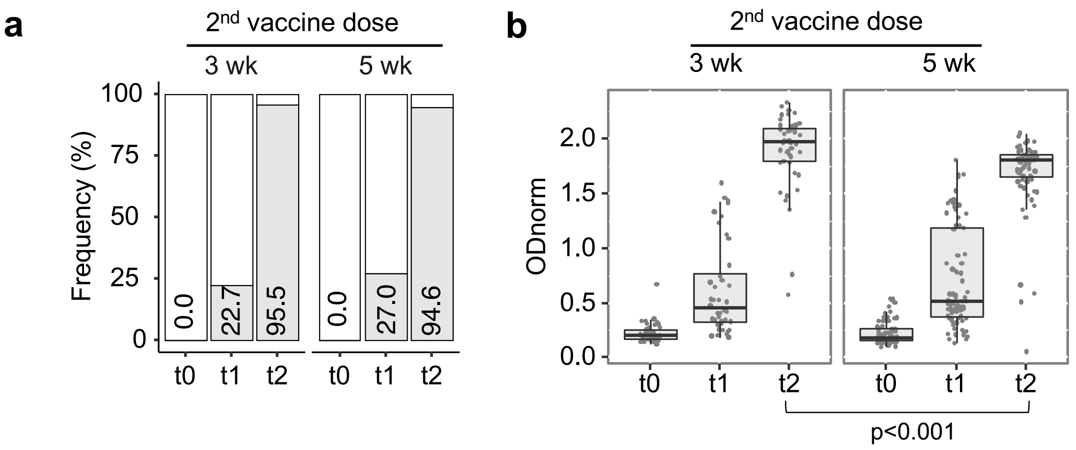

c

	t0	t1	t2
**3 weeks (n=44)**			
median [IQR]	0.21 [0.17-0.26]	0.46 [0.33-0.77]	1.97 [1.79-2.09]
Positivity; n (%; 95% CI)	0 (0.0; 0.0-0.1)	10 (22.7; 12.8-37.0)	42 (95.5; 84.9-98.7)
**5 weeks (n=74)**			
median [IQR]	0.19 [0.16-0.27]	0.52 [0.37-1.18]	1.81 [1.65-1.86]
Positivity; n (%; 95% CI)	0 (0.0; 0.0-0.05)	20 (27.0; 18.2-38.1)	70 (94.6; 86.9-97.9)

Supplementary Figure S1: Effect of delayed administration of 2nd vaccine dose in the NHR cohort. The 2nd vaccine dose was administered 3 weeks (n=44 participants), or 5 weeks (n=74 participants) after the 1st dose. Shown are reanalysis of the data for anti- spike IgG (ELISA) presented in Fig.1C, now partitioned according to the time to the 2nd vaccine dose in weeks (wk). **a)** Seroconversion defined by frequency of samples testing positive (grey bars) at the indicated time point. Respective values are indicated inside each bar**. b)** Semi-quantitative measurements. Data points represent individual participants, boxes denote interquartile range, horizontal line represent the median, and whiskers denote the minimum and maximum values below or above the median at 1.5 times the interquartile range. Wilcoxon rank sum test, two-sided, revealed a significant difference between groups at t2 p=$2.7927 \times 10^{-05,}$ but not at t1 p=0.32340e. **c**) Table displaying anti-spike IgG ODnorm (Median and [IQR]) and positivity, and measured at t0, t1 and t2. Source data are provided as a Source Data file.

Supplementary Figure S2.

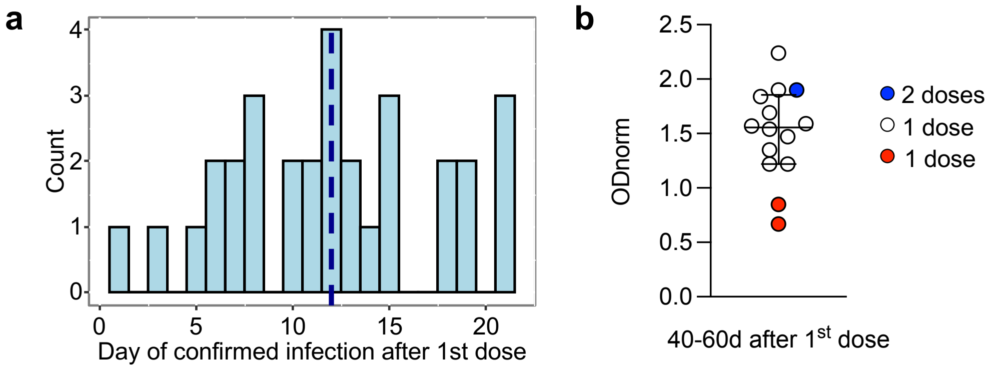

c

**Sex, % (n)**	
male	22.6% (7/31)
female	77.4% (24/31)
**Age, median [range]**	36 years [22-63]
**Anti-spike IgG**	
Positivity, % (n)	85.7% (12/14)
ODnorm, median [IQR]	1.55 [1.22-1.85]

Supplementary Figure S2. Infections after the 1st dose. 31 participants were diagnosed COVID-19 after the 1st vaccination, 14/31 contributed sera post-infection. **a)** Histogram of participants (n=31) who got infected with SARS-CoV-2 after the 1st vaccine dose, as determined by a positive qPCR test between t0 and t1. Dashed line represents median day of infection after 1st dose (t0). 24/31 (77.4%) of the infected participants, were diagnosed in the first 15 days after the 1st dose. **b)** Anti-spike IgG ODnorm measured 40-60 days after the 1st vaccine dose in participants infected during the first 4 weeks after receiving the 1st dose (n=14). Data points represent individual participants, major horizontal line represent the median, and whiskers denote interquartile range. Highlighted are 2 non-responders (red circles), and 1 participant who received 2 vaccine doses (blue circle). **c)** Table indicating sex and age distribution (upper part), and anti-spike IgG positivity, as well as ODnorm median and [IQR], both measured 40-60 days after the 1st dose (lower part). Source data are provided as a Source Data file.
