# Supplementary material for: Population homogeneity for the antibody response to COVID-19 BNT162b2/Comirnaty vaccine is only reached after the second dose across all adult age ranges
Source: Nat Commun. 2022 Jan 10;13:140. doi: 10.1038/s41467-021-27761-z (PMC8748650; doi:10.1038/s41467-021-27761-z)
Supplement: Supplementary file 3 — Reporting Summary [file 41467_2021_27761_MOESM3_ESM.pdf]

nature research

Corresponding author(s): Jocelyne Demengeot

Last updated by author(s): November 16, 2021

Reporting Summary

Nature Research wishes to improve the reproducibility of the work that we publish. This form provides structure for consistency and transparency in reporting. For further information on Nature Research policies, see our Editorial Policies and the Editorial Policy Checklist.

Statistics

For all statistical analyses, confirm that the following items are present in the figure legend, table legend, main text, or Methods section.

n/a	Confirmed	
☐	☒	The exact sample size (*n*) for each experimental group/condition, given as a discrete number and unit of measurement
☐	☒	A statement on whether measurements were taken from distinct samples or whether the same sample was measured repeatedly
☐	☒	The statistical test(s) used AND whether they are one- or two-sided<br>*Only common tests should be described solely by name; describe more complex techniques in the Methods section.*
☐	☒	A description of all covariates tested
☐	☒	A description of any assumptions or corrections, such as tests of normality and adjustment for multiple comparisons
☐	☒	A full description of the statistical parameters including central tendency (e.g. means) or other basic estimates (e.g. regression coefficient) AND variation (e.g. standard deviation) or associated estimates of uncertainty (e.g. confidence intervals)
☐	☒	For null hypothesis testing, the test statistic (e.g. *F*, *t*, *r*) with confidence intervals, effect sizes, degrees of freedom and *P* value noted<br>*Give P values as exact values whenever suitable.*
☒	☐	For Bayesian analysis, information on the choice of priors and Markov chain Monte Carlo settings
☒	☐	For hierarchical and complex designs, identification of the appropriate level for tests and full reporting of outcomes
☒	☐	Estimates of effect sizes (e.g. Cohen's *d*, Pearson's *r*), indicating how they were calculated

Our web collection on statistics for biologists contains articles on many of the points above.

Software and code

Policy information about availability of computer code

Data collection	No software was used to collect the data, except that from a standard Elisa reader
Data analysis	Statistical analyses and data representation (figures) were carried out using R ((r-project.org, version 4.0.4 GUI 1.74), R studio (version 1.1.463) and the main package ggplot2 (version 3.3.5). References provided in supplemental material.

For manuscripts utilizing custom algorithms or software that are central to the research but not yet described in published literature, software must be made available to editors and reviewers. We strongly encourage code deposition in a community repository (e.g. GitHub). See the Nature Research guidelines for submitting code & software for further information.

Data

Policy information about availability of data

All manuscripts must include a data availability statement. This statement should provide the following information, where applicable:

- Accession codes, unique identifiers, or web links for publicly available datasets
- A list of figures that have associated raw data
- A description of any restrictions on data availability

All data necessary to interpret, verify and extend the research in the article, and presented in Figures 1 to 5, supplementary Figure 1 and supplementary Figure 2, is provided in the “source data” file.

Field-specific reporting

Please select the one below that is the best fit for your research. If you are not sure, read the appropriate sections before making your selection.

☒ Life sciences ☐ Behavioural & social sciences ☐ Ecological, evolutionary & environmental sciences

For a reference copy of the document with all sections, see nature.com/documents/nr-reporting-summary-flat.pdf

Life sciences study design

All studies must disclose on these points even when the disclosure is negative.

Sample size

The sample size was not predicted in advance; participants were recruited using a non-probabilistic method by convenience sampling of health professionals and nursing home residents who volunteered to participate. The effect size (Cohen's f, calculated on IgG levels in 5 age groups of the HCW cohort) estimates a minimum group size of n=35 to detect a mean group difference of 0.1 in ODnorm, with a significance level of 0.05 and a power of 80%.

Data exclusions

No data were excluded, though specific analysis were restricted: e.g. Diagnosed and inferred cases of COVID-19 post t0 were excluded from the main immunogenicity analysis, as were samples from participants who did not participated to the three collection time points.

Replication

Frequency of seroconversion (positivity), anti-spike levels and age effects on these, were successfully replicated with two different immunoassays using two different target antigens: ELISA to quantify IgG, IgM and IgA anti-full-length spike and Electro-chemiluminescence immunoassay (ECLIA) to quantify Ig anti-RBD
(Elecsys® Anti-SARS-CoV2 S, Roche). Anti-full-length spike measurements were performed in duplicates, and rare discrepancies between replicates were resolved through repeated duplicate measurements. Data from positive and negative control samples included in each run were reproducible across assays.

Randomization

Randomization was not applicable to our study as we conducted an observational study without intervention. The criterion of enrollment in this study was individuals who initiated vaccination with BNT162b2 and volunteered to participate.

Blinding

All data from immunoassays was generated prior to information on participants sex and age.

Reporting for specific materials, systems and methods

We require information from authors about some types of materials, experimental systems and methods used in many studies. Here, indicate whether each material, system or method listed is relevant to your study. If you are not sure if a list item applies to your research, read the appropriate section before selecting a response.

Materials & experimental systems

n/a	Involved in the study
☐	☒ Antibodies
☒	☐ Eukaryotic cell lines
☒	☐ Palaeontology and archaeology
☒	☐ Animals and other organisms
☐	☒ Human research participants
☒	☐ Clinical data
☒	☐ Dual use research of concern

Methods

n/a	Involved in the study
☒	☐ ChIP-seq
☒	☐ Flow cytometry
☒	☐ MRI-based neuroimaging

Antibodies

Antibodies used

Goat Anti-Human IgG Fc (HRP) from abcam, Cat# ab97225; Goat Anti-Human IgM mu chain (HRP) from abcam, Cat# ab97205; Goat Anti-Human IgA alpha chain (HRP) from Abcam, Cat# ab97215

Validation

We optimized the reccomended concentrations for these antibodies and validated the assay performance by testing 1000 pre-pandemic sera and 40 COVID-19 patients diagnosed at least 10 days prior to sera collection. ROC curve analysis determined a specificity of 99.3%, 99.2%, 99.2%, and a sensitivity of 95.9%, 61.2% and 73.7% for IgG, IgM and IgA, respectively.
From abcam manufacturer:
IgG Specificity: By immunoelectrophoresis and ELISA this antibody reacts specifically with Human IgG. Cross reactivity with other immunoglobulins and light chains is less than 0.1%.
IgM Specificity: By immunoelectrophoresis and ELISA this antibody reacts specifically with Human IgM. Cross reactivity with other immunoglobulins and light chains is less than 0.1%.
IgA Specificity: By immunoelectrophoresis and ELISA this antibody reacts specifically with Human IgA. Cross reactivity with other immunoglobulins and light chains is less than 0.1%.

Human research participants

Policy information about studies involving human research participants

Population characteristics	The study followed 1245 healthcare workers (HCW cohort) and 146 nursing home residents (NHR cohort). Both cohorts present a biased sex ratio (females 79% in HCW and 74% in NHR), as is common in these populations in occident, and together encompass a broad age range (median [age range]: HCW 43 [19-70] and NHR 87 [70-99])
Recruitment	The criterion of enrollment in this study was individuals who initiated vaccination with BNT162b2 and volunteered to participate. Enrollement was associated in time and space with administration of the 1st dose of BTN162b2, made a National priori with close to full adhesion in the two target populations (Health care workers and Nursing home resident). Self-selection was unlikely to bias the results of this study. Over-representation of female participants is consistent with the demographic of each target population, and the data analysis includes sex partitioning alleviating the potential impact of this bias.
Ethics oversight	The study was approved by the Ethics committees of the Centro Hospitalar Lisboa Ocidental and the Administração Regional de Lisboa e Vale do Tejo, in compliance with the Declaration of Helsinki, and follows international and national guidelines for health data protection. All participants provided informed consent to take part in the study

Note that full information on the approval of the study protocol must also be provided in the manuscript.
